# Plant-Mediated Soil Sickness: Steering the Rhizosphere into a Pathogenic Niche

**DOI:** 10.3390/microorganisms14010052

**Published:** 2025-12-25

**Authors:** Jichao Li, Mingju Qi, Jinyu Zhang, Yingmei Zuo

**Affiliations:** Medicinal Plants Research Institute, Yunnan Academy of Agricultural Sciences, No. 2238 Beijing Road, Kunming 650221, China

**Keywords:** root exudates, soil microbiome, plant–soil feedback, consecutive monoculture problem, *Fusarium oxysporum*

## Abstract

Continuous monoculture of *Panax notoginseng* leads to severe replant disease, yet the mechanisms by which root exudates mediate rhizosphere microbiome assembly and pathogen enrichment remain poorly understood. Here, we demonstrate that long-term root exudate accumulation acts as an ecological filter, driving the fungal community toward a phylogenetically impoverished, pathogen-dominated state. Specifically, exudates enriched the soil-borne pathogen *Fusarium* while reducing the abundance of potentially antagonistic fungi. In contrast, bacterial communities exhibited higher resilience, with exudates selectively enriching oligotrophic taxa such as *Terrimonas* and *MND1*, but suppressing nitrifying bacteria (e.g., *Nitrospira*) and plant-growth-promoting rhizobacteria (PGPR). Microbial functional profiling revealed a shift in nitrogen cycling, characterized by suppressed nitrification and enhanced nitrate reduction. Crucially, co-occurrence network analysis identified bacterial taxa strongly negatively correlated with *Fusarium*, providing a synthetic community blueprint for biocontrol strategies. Our study establishes a mechanistic link between root exudate accumulation and negative plant–soil feedback in monoculture systems, highlighting microbiome reprogramming as a key driver of replant disease. These insights offer novel avenues for manipulating rhizosphere microbiomes to sustain crop productivity in intensive agricultural systems.

## 1. Introduction

In the mountainous regions of China, *Panax notoginseng*, a herbaceous perennial valued in traditional Chinese medicine “as precious as gold”, faces a silent survival crisis. This plant, renowned for its dried roots and rhizomes, confronts a severe ecological challenge known as consecutive monoculture problem (CMP) [1]. Data reveal a dramatic decline in survival rates: below 50% after four years of initial cultivation, and a further plunge to less than 10% when replanted within a year post-harvest [2]. This pervasive issue, common among many perennial medicinal plants, critically threatens the sustainable supply of medicinal herbs, making the elucidation of its mechanisms and the development of mitigation strategies an urgent scientific priority [3].

A sophisticated “microbial immune system”, dominated by beneficial bacteria such as *Pseudomonas* and *Burkholderia*, operates within the healthy rhizosphere of *P. notoginseng*. These bacteria form a robust biological defense line through intense resource competition and the secretion of antimicrobial compounds, effectively suppressing the invasion of soil-borne pathogens like *Fusarium oxysporum* [4]. However, under consecutive monoculture conditions, this innate barrier undergoes systematic collapse, allowing pathogens to breach the defenses and trigger devastating root rot [1].

What dismantles this crucial microbial barrier? Mounting evidence indicates that the crisis originates from disrupted dialogue between the plant and its soil microbiome. The root system of *P. notoginseng* serves not merely as a reservoir of bioactive compounds but as a dynamic ecological interface. It engages in intricate chemical communication with root-associated microorganisms through rhizodeposits, including root exudates [5]. These exudates, rich in secondary metabolites such as specific saponins and phenolic acids, exhibit allelopathic autotoxicity that directly inhibits the growth of subsequent plants. Simultaneously, they function as crucial nutritional substrates, actively shaping the structure and composition of the rhizosphere microbial community [6].

Critically, the concentration of these root exudates may act as an “ecological switch” dictating the fate of the microbial assemblage. At lower concentrations, they likely sustain a balanced, bacterial-dominated community enriched with plant-growth-promoting rhizobacteria (PGPR), thereby supporting ecosystem stability [7]. However, beyond a critical threshold progressively reached under continuous monoculture, these accumulations trigger a qualitative shift in the microbiome. This transition towards a fungal-dominated state facilitates the invasion and proliferation of soil-borne pathogens like *F. oxysporum*, culminating in widespread root rot and a systemic impact on ecosystem functions [2]. Preliminary studies have indicated that the long-term cultivation of *Panax notoginseng* can lead to a gradual accumulation of rhizosphere pathogens [8]. Despite this, how and whether root exudate accumulation specifically translates into the negative plant–soil feedback that drives this accumulation process remains poorly understood. This knowledge gap significantly impedes a mechanistic understanding of its role in disease onset and the development of sustainable solutions to mitigate soil sickness.

Positioned at this scientific frontier, our research employs root exudates as a strategic entry point to dissect the mechanisms driving this ecological failure. By manipulating their accumulation dynamics, we aim to answer three questions: (1) Whether and how the accumulation of root exudates acts as a chemical driver, leading to the reassembly of the rhizosphere microbiome. (2) Whether this reassembly constitutes a deterministic negative plant–soil feedback that triggers a pathogenic shift and specifically enriches the *F. oxysporum*. (3) To identify the specific keystone bacterial and fungal taxa most susceptible to this perturbation.

## 2. Materials and Methods

### 2.1. Study Site Description

The experiment was conducted at the experimental station of Yunnan Academy of Agricultural Sciences in Kunming, Yunnan Province, China (25°02′15.0″ N, 102°43′20.0″ E). The site is located in a low-latitude plateau area with a subtropical monsoon humid climate. The mean annual temperature is 16 °C, with the coldest months being January and December (average temperature 9 °C) and the warmest months being June and July (average temperature 21 °C). The mean annual precipitation is 77.75 mm (data retrieved from China Weather Network). A greenhouse with a height of 3 m was built at this site; its exterior was covered with a single layer of polyethylene white film, and the interior was equipped with three layers of black shading nets that provided approximately 75% shading. This facility was designed to achieve shading, rain protection, and prevention of damage caused by birds and insects. The experimental field consisted of a weakly acidic (pH 6.5) red soil (Alisol), with an organic matter content of 5.0% and containing total nitrogen (0.30%), available phosphorus (35 mg kg^−1^), and available potassium (175 mg kg^−1^).

### 2.2. Experimental Materials

The test soil was uncultivated red soil with no prior cultivation history of *P. notoginseng*, collected from a cultivation base in Wenshan City, Yunnan Province, China. The test materials were one-year-old *P. notoginseng* seedlings, which were raised from disinfected seeds in a nursery substrate. All seedlings were robust, plump, and free from pests and diseases.

### 2.3. Root Exudate Collection Method

Root exudates were collected using a substrate-based cultivation system, with perlite serving as the inert and hydrophilic substrate. Seedlings were transplanted into 32-cell trays, where the bottom of each cell was sealed with a 2-mm nylon mesh and filled with perlite. Ten replicate groups were set up, with one seedling planted per cell. The trays were placed in square containers containing 2 cm of deionized water at the bottom, and the deionized water was replenished every two days. After seedling emergence, foliar fertilizer was applied weekly; during fertilization, a plastic film was laid over the perlite to avoid contamination of the substrate.

Root exudate collection was initiated one month after seedling emergence. The perlite in each cell was thoroughly rinsed with deionized water three times, with 20 mL of water used for each rinse, and this rinsing process was performed weekly. The leachate collected from the containers was filtered through a 0.45 µm membrane and then concentrated from 1000 mL to 50 mL under reduced pressure at 50 °C. The concentrated solution was immediately freeze-dried into powder and stored at −20 °C for subsequent experimental use.

### 2.4. Experimental Design

A pot experiment was carried out in the greenhouse described in Section 2.1. Disinfected *P. notoginseng* seedlings were transplanted into pots filled with the aforementioned red soil, with three seedlings per pot. After covering the roots with soil to a level just below the shoot base, the seedlings were irrigated with tap water of equal volume every week. One month later, seedlings with uniform growth status were selected and divided into four treatment groups, with three replicates per group and 70 seedlings in each treatment. The specific grouping was as follows: Group A was treated with root exudates every 7 days for a total of 49 days, with 7 applications in total; Group B was treated with root exudates every 21 days for a total of 147 days, also with 7 applications in total. Meanwhile, two corresponding control groups (Ack and Bck) were set up, which were irrigated with deionized water following the same schedules as Groups A and B, respectively. Each application involved administering 50 mL of root exudate solution per seedling, which was equivalent to the amount of root exudates collected from two seedlings over 7 days. All other management measures were kept consistent across all groups throughout the experiment.

### 2.5. Sampling

Rhizosphere soil samples were collected at different time points. For Groups S and Sck, samples were collected before the start of treatment (labeled S0 and Sck0, respectively) and after 49 days of treatment (labeled S6 and Sck6, respectively). For Groups L and Lck, samples were collected before treatment (labeled L0 and Lck0, respectively) and after 126 days of treatment (labeled L6 and Lck6, respectively). In total, eight sample groups (S0, S6, Sck0, Sck6, L0, L6, Lck0, Lck6) were obtained. Third-generation sequencing technology was used to perform full-length bacterial 16S rRNA gene sequencing and fungal internal transcribed spacer (ITS) sequencing on these samples. The sampling procedure was conducted as follows: Ten seedlings were randomly selected from each group. The surface soil was removed first, and then the seedlings were carefully uprooted using a sterilized shovel. After shaking off the loosely attached soil, the rhizosphere soil tightly adhering to the root surfaces was brushed off with a sterile brush and collected. A total of 5–10 g of soil was sampled from multiple random positions, homogenized thoroughly, and placed into sterile plastic bags marked with sample IDs and sampling dates. The samples were transported to the laboratory in an ice box. After removing gravel and plant debris, the soil samples were ground and sieved through a 2 mm mesh sieve. The processed soil samples were aliquoted into 2 mL cryotubes and stored at −80 °C until subsequent analysis.

### 2.6. Microbiome Analysis

Total genomic DNA was extracted from soil samples using the QIAGEN DNeasy PowerSoil Pro Kit (Cat. No. 47014) according to the manufacturer’s protocol. The full-length bacterial 16S rRNA gene (V1–V9 regions) and the fungal ITS region were amplified with barcoded primers: 27F (5′-AGRGTTTGATYMTGGCTCAG-3′) and 1492R (5′-TACCTTGTTACGACTT-3′) for bacteria, and ITS1F (5′-CTTGGTCATTTAGAGGAAGTAA-3′) and ITS4 (5′-TCCTCCGCTTATTGATATGC-3′) for fungi. PCR was performed in 20 µL reactions containing 0.5 U of KOD FX Neo high-fidelity DNA polymerase, 1× buffer, 200 µM dNTPs, 0.12 µM of each primer, and approximately 5 ng of template DNA. Thermal cycling conditions were: 94 °C for 2 min; 30 cycles of 98 °C for 10 s, 55 °C for 30 s, and 68 °C for 1.5 min; and a final extension at 72 °C for 5 min. Nine independent biological replicates were included per treatment group. Amplicons were purified, pooled in equimolar amounts, and used to construct sequencing libraries following the PacBio SMRTbell Express Template Prep Kit 2.0 protocol. Libraries were sequenced on a PacBio Sequel II platform using single-molecule real-time (SMRT) technology.

### 2.7. Statistical and Bioinformatic Analysis

High-accuracy Circular Consensus Sequences (CCS) were generated from raw polymerase reads (subreads) using the ccs tool (SMRT Link suite, Pacific Biosciences, Menlo Park, CA, USA). Primer trimming and demultiplexing by unique barcodes were performed with the lima tool. Primer-trimmed CCS reads were then denoised using the DADA2 algorithm, which models sample-specific error profiles to distinguish true biological variation from PCR and sequencing errors, thereby inferring exact amplicon sequence variants (ASVs) at single-nucleotide resolution. Chimeric sequences were subsequently identified and removed using DADA2’s removeBimeraDenovo function. Representative ASV sequences were taxonomically classified against the SILVA database (release 138) for bacterial 16S rRNA genes and the UNITE database (release 9.0) for fungal ITS regions. The final ASV abundance table (ASVs × biological replicates) served as the exact count matrix for all downstream ecological and statistical analyses. Taxonomic annotation was performed at the phylum, class, order, family, genus, and species levels by aligning the high-quality sequences against reference databases (e.g., the SILVA database for 16S rRNA genes and the UNITE database for ITS regions) for species classification, and phylogenetic trees were constructed accordingly. Alpha diversity (representing within-sample diversity, evaluated using indices such as Chao1 and Shannon) and beta diversity (representing between-sample diversity, evaluated using metrics including weighted/unweighted UniFrac distances and Bray-Curtis distances) were analyzed. Principal coordinates analysis (PCoA) was performed based on the beta diversity matrices. The Wilcoxon rank-sum test was used to analyze the differences in alpha diversity indices among groups, while the linear discriminant analysis effect size (LEfSe) algorithm was employed to identify differentially abundant microbial taxa. Additionally, the Metastats method was adopted to further verify the significance of the differentially abundant microbial features. A random forest model was established to screen out the most predictive microbial taxa for distinguishing between different sample groups. Fungal functional guilds were predicted using the FunGuild database, which associates fungal taxonomic information with ecological functional categories through a bioinformatics annotation pipeline. Furthermore, co-occurrence networks of microbial communities were constructed based on reliable correlation matrices (e.g., SparCC), and the networks were visualized using the R package visNetwork (v2.1.0). All statistical analyses and visualization works were implemented in R software (v3.6.1) with the assistance of multiple packages, including psych (v2.1.9) and igraph (v1.2.5).

## 3. Results

### 3.1. Root Exudate Accumulation Drives Pathogenic Restructuring of the Rhizosphere Fungal Community

To determine whether, and to what extent, the accumulation of *Panax notoginseng* root exudates directs the assembly and succession of the rhizosphere fungal community. We established a gradient of exudate exposure, comparing treatments receiving root exudates against control groups receiving sterile water. Furthermore, we contrasted the initial community state with its state following both short-term (seven applications over 49 days) and long-term (seven applications over 127 days) exudate exposure to dissect the temporal dynamics of microbial succession.

**Figure 1:**
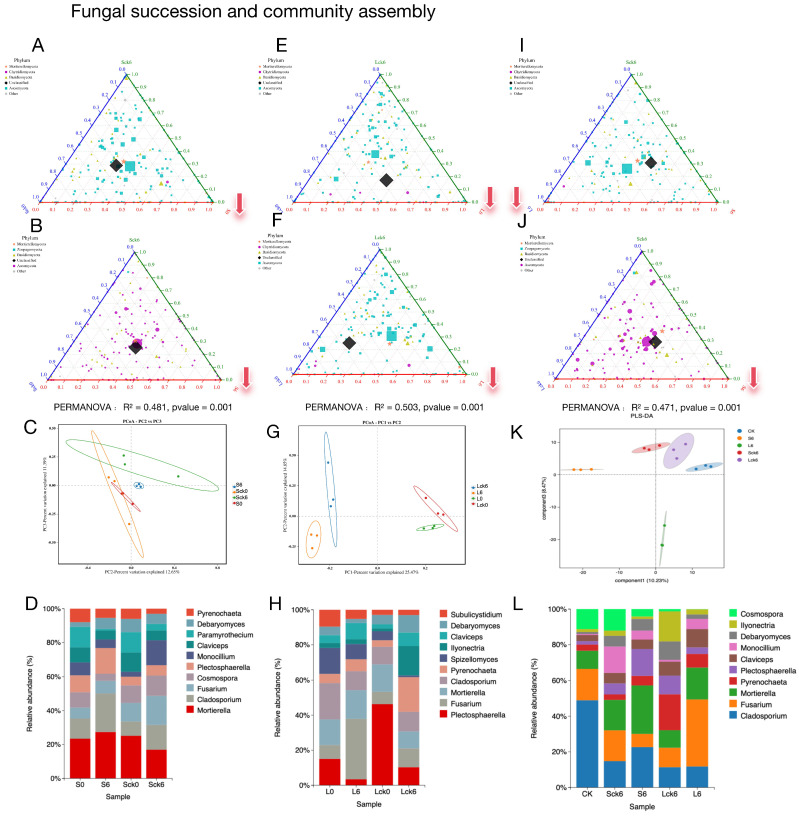
Root exudate accumulation drives fungal community succession and pathogenic restructuring in the rhizosphere. (**A**–**D**) Short-term exudate effects. (**A**,**B**) Fungal community structure in control (untreated), short-term exudate-amended, and sterile-water-amended rhizosphere samples. (**C**) Principal components analysis (PCA) illustrating the separation of fungal communities under different treatments. (**D**) Species distribution and relative abundance of the top 10 fungal genera. (**E**–**H**) Long-term exudate effects. (**E**,**F**) Fungal community structure following long-term exudate amendment compared to control and sterile-water groups. (**G**) PCA ordination demonstrating the distinct trajectory of fungal assembly under sustained exudate exposure. (**H**) Shift in the distribution of dominant fungal genera. (**I**–**L**) Temporal dynamics of exudate-induced restructuring. (**I**,**J**) Comparative community structure between short-term and long-term exudate treatments. (**K**) PCA highlighting the divergence in fungal communities over time. (**L**) Key fungal taxa that were differentially abundant between short- and long-term regimes, including the marked enrichment of the pathogen *Fusarium*. Note: Sck0/SCK6: soil before/after 49-day sterile water amendment; S0/S6: soil before/after 49-day root exudate amendment; Lck0/LCK6: soil before/after 127-day sterile water amendment; L0/L6: soil before/after 127-day root exudate amendment; CK: baseline soil.

Root exudates reconfigure fungal community assembly and reduce diversity. Comparative analysis of exudate-amended versus sterile-water-amended soils revealed a significant divergence in fungal community structure (Figure 1A,B,E,F). Counter to the hypothesis that exudates might serve as a general nutrient subsidy promoting microbial proliferation, their addition consistently resulted in a reduction of fungal richness, particularly affecting taxa within the Ascomycota phylum (Figure 1A,B,E,F). This exudate-driven restructuring is further evidenced by a clear separation of treatment groups in principal component analysis (Figure 1C,G) and shifts in the relative abundance of the top 10 fungal genera (Figure 1D,H).

Exudate accumulation redirects ecological succession toward a depauperate state. Analysis of temporal shifts revealed that the trajectory of fungal succession is fundamentally altered by root exudates. While control communities receiving sterile water developed toward greater taxonomic richness, this pattern was amplified over the long term and marked by an increase in *Ascomycota*, and the exudate-amended soils followed a divergent path. Both short- and long-term exposure to exudates drove the community toward a less diverse state (Figure 1A,B,E,F).

Long-term exudate accumulation selectively enriches pathogenic taxa. A direct comparison of short- versus long-term exposure pinpointed the cumulative impact of exudates. The long-term treatment specifically fostered the enrichment of the *Mortierellomycota* and *Chytridiomycotaphyla*, whereas the short-term treatment favored a higher relative abundance of *Ascomycota* (Figure 1L). Critically, principal component analysis confirmed distinct community profiles between these two time points (Figure 1K), with the long-term state being defined by a significant enrichment of the pathogen *Fusarium* (Figure 1L).

In summary, our results demonstrate that the long-term accumulation of *P. notoginseng* root exudates exert a profound and negative influence on the resident fungal community, effectively steering its assembly and succession. The data confirm our central hypothesis: the continuous release of root exudates creates a soil environment that is selectively favorable for the enrichment of soil-borne pathogens like *Fusarium*. This pathogen-tropic shift, driven by the host plant’s own exudates, represents a critical mechanistic link in the onset of root rot and the broader phenomenon of consecutive monoculture problems. Our findings position root exudate accumulation as a pivotal factor in mediating the negative plant–soil feedback that undermines sustainable cultivation.

### 3.2. Root Exudate Accumulation Drives Rhizosphere Enrichment of the Soil-Borne Pathogen Fusarium

The enrichment of soil-borne pathogenic fungi within the genus *Fusarium* in the rhizosphere is a primary agent leading to root rot and consecutive monoculture problem (CMP). To determine whether the accumulation of root exudates acts as a key factor in attracting and facilitating the colonization of *Fusarium*, which thereby exacerbates disease and CMP, we analyzed differential microbial taxa across treatments and specifically assessed the significance and relative abundance of *Fusarium*.

**Figure 2:**
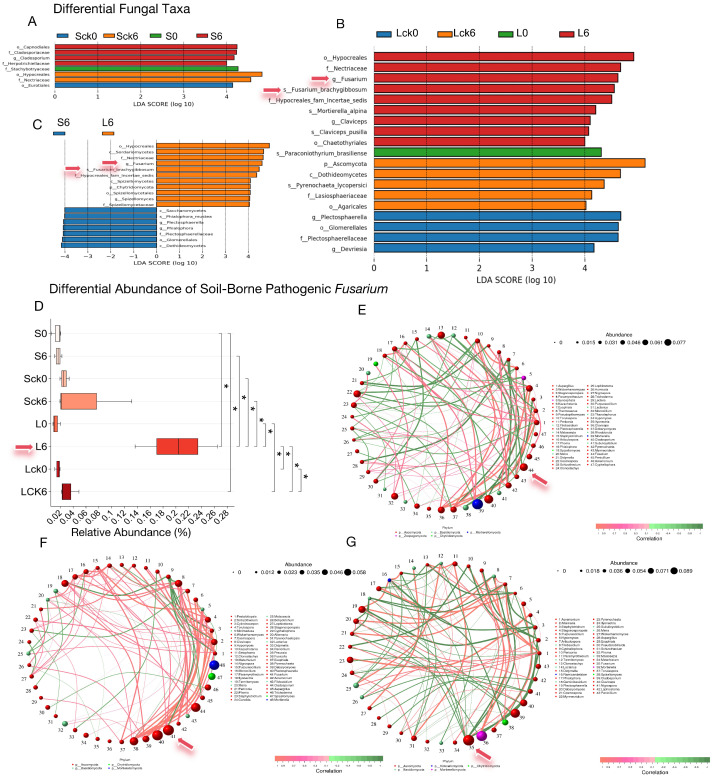
Fungal differential abundance and *Fusarium* enrichment in response to root exudate accumulation. (**A**) Differentially abundant fungal taxa under short-term exudate amendment, identified by LEfSe analysis. (**B**) Differentially abundant taxa under long-term exudate amendment. The genus *Fusarium* (including *F. brachygibbosum*) emerged as a statistically significant indicator in long-term exudate-treated soils, distinguishing them from all other treatments. (**C**) Comparison between short- and long-term exudate treatments reveals that prolonged accumulation of root exudates significantly enriched *Fusarium* and *F. brachygibbosum* in the rhizosphere. (**D**) Relative abundance of *Fusarium* across treatments, demonstrating its specific enrichment in long-term exudate-amended soils compared to sterile water controls, untreated, and short-term exudate groups. This indicates that sustained root exudation is a critical factor driving pathogen enrichment and ultimately contributes to negative plant-soil-microbe feedback and replant disease. (**E**–**G**) Co-occurrence network analysis of fungi (correlation coefficient > 0.8, *p* < 0.05) in (**E**) sterile water-amended, (**F**) pre-exudate, and (**G**) short-versus long-term exudate-amended soils. In each network, *Fusarium* acts as a highly connected and abundant keystone node. Networks also identify taxa significantly negatively correlated with *Fusarium*, offering a functional basis for inferring microbial interactions and designing synthetic biocontrol communities. Note: Sck0/SCK6: soil before/after 49-day sterile water amendment; S0/S6: soil before/after 49-day root exudate amendment; Lck0/LCK6: soil before/after 127-day sterile water amendment; L0/L6: soil before/after 127-day root exudate amendment. * *p* < 0.05 by two-tailed Student’s *t*-test *.

Our findings reveal that only in the long-term root exudate treatment was *Fusarium*, including the species *F. brachygibbosum*, identified as a significantly enriched taxon (Figure 2A–C). Boxplot analysis of its relative abundance confirmed a significant increase (Figure 2D, *p* < 0.05) in the long-term exudate group, which markedly differed from both the short-term exudate and long-term sterile water control groups (Figure 2D, *p* < 0.05), indicating that sustained exudate accumulation selectively enriches *Fusarium* in the rhizosphere.

Correlation network analysis further positioned *Fusarium* as a highly connected hub with substantial relative abundance (Figure 2E–G). To inform the construction of a synthetic biocontrol community, we identified fungal taxa exhibiting significant negative correlations with *Fusarium*. Intriguingly, these potential antagonists predominantly belonged to the same phylum, Ascomycota, and included genera such as *Articulospora*, *Plectosphaerella*, *Debaryomyces*, *Cosmospora*, *Acremonium*, and *Hypomyces* (Figure 2E–G).

In summary, the long-term accumulation of *P. notoginseng* root exudates selectively enriches pathogenic *Fusarium* in the rhizosphere, positioning it as a critical driver in the of root rot and the establishment of CMP. Consequently, future biocontrol strategies should prioritize microbial candidates capable of degrading or neutralizing these host-specific exudates, thereby intercepting pathogen recruitment and reprogramming the rhizosphere microbiome toward a disease-suppressive state.

### 3.3. Root Exudate Accumulation Differentially Modulates Rhizosphere Bacterial Community Succession and Assembly

**Figure 3:**
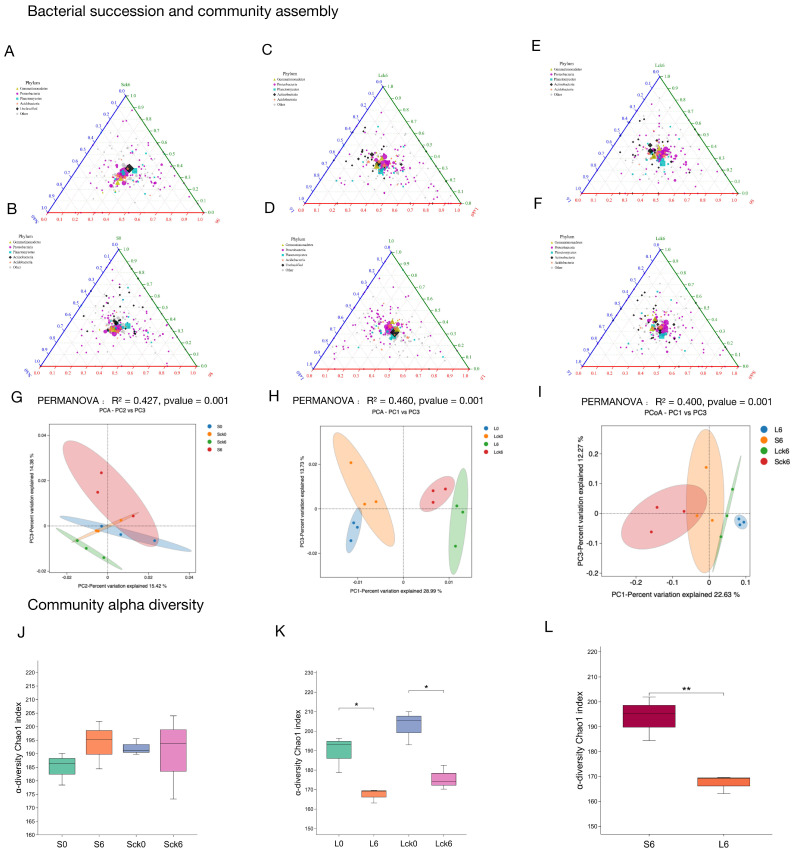
Root exudate accumulation drives bacterial community succession in the rhizosphere. (**A**,**B**) Short-term exudate effects. Bacterial community structure in control (untreated), short-term exudate-amended, and sterile-water-amended rhizosphere samples. (**C**,**D**) Bacterial community structure following long-term exudate amendment compared to control and sterile-water groups. (**E**,**F**) Comparative community structure between short-term and long-term exudate treatments. (**G**–**I**) Principal components analysis (PCA) illustrating the separation of bacterial communities under different treatments. (**G**) Short-term exudate effects, (**H**) short-term exudate effects, (**I**) comparative community structure between short-term and long-term exudate treatments. (**J**–**L**) Comparison of microbial alpha diversity (Chao index) across different bacterial communities. (**J**) Short-term exudate effects, (**K**) short-term exudate effects, (**L**) comparative community structure between short-term and long-term exudate treatments. Long-term root exudate amendment reduced bacterial community diversity compared to short-term treatment. Sample abbreviations follow definitions in Figure 1 and Figure 2. * *p* < 0.05, ** *p* < 0.01 (two-tailed Student’s *t*-test).

While our findings demonstrate that long-term root exudate accumulation drives significant restructuring of the fungal microbiome, its effect on the functionally broader bacterial community remained unclear. Here, we report that root exudates did not induce significant shifts in overall bacterial community structure or diversity indices, though subtle taxonomic redistribution was observed under prolonged exposure. Notably, continuous monoculture of *P. notoginseng* itself led to a consistent decline in bacterial diversity across treatments (Figure 3K,L).

Root exudates selectively enrich Proteobacteria without altering global diversity (Figure 3A–D). Comparative analysis between exudate-amended and sterile-water-treated soils revealed that root exudates consistently enriched members of the Proteobacteria phylum (Figure 3A–D). Principal coordinates analysis indicated that exudate-driven divergence in bacterial assembly was detectable only after long-term treatment, with no significant community-wide shift observed in the short term (Figure 3G,H). Furthermore, alpha diversity indices showed no statistically significant differences between long-term exudate and control groups (Figure 3J–L), suggesting that exudates reshape bacterial composition within a conserved diversity framework.

Temporal bacterial succession is marked by progressive Proteobacterial enrichment and diversity loss. Analysis of succession trajectories from pre- to post-exudate exposure showed that both short- and long-term treatments led to similar directional shifts: a consistent increase in Proteobacteria and a concurrent reduction in diversity (Figure 3A–D). However, while short-term treatment did not significantly alter overall community structure, long-term exposure resulted in discernible divergence (Figure 3G,H).

Long-term exudation drives compositional divergence from short-term communities. Direct comparison between short- (49-day) and long-term (127-day) bacterial profiles revealed that extended exudate accumulation led to a relative reduction in Proteobacteria compared to the short-term group (Figure 3E,F). This shift contributed to a clear separation between the two time points in principal coordinates space, highlighting duration-dependent effects of root exudates on bacterial assembly (Figure 3I).

In contrast to the pronounced restructuring observed in the fungal microbiome, root exudates exerted a more subtle influence on bacterial communities, primarily modulating relative taxon abundances rather than inducing marked changes in richness or global community structure. These results underscore a kingdom-specific response to plant-derived biochemical cues and emphasize the critical role of exposure duration in shaping rhizosphere bacterial dynamics. The limited bacterial restructuring despite strong fungal rearrangement suggests that fungal communities may serve as earlier and more sensitive indicators of plant–soil feedback in monoculture systems, with important implications for targeted microbiome management in sustainable agriculture.

### 3.4. Root Exudate Accumulation Alters Microbial Functional Profiles in the Rhizosphere

Changes in bacterial community structure can profoundly influence ecosystem functioning. To assess whether long-term root exudate accumulation alters the functional potential of the rhizosphere microbiome, we performed functional annotation and compared metabolic profiles between treatments.

**Figure 4:**
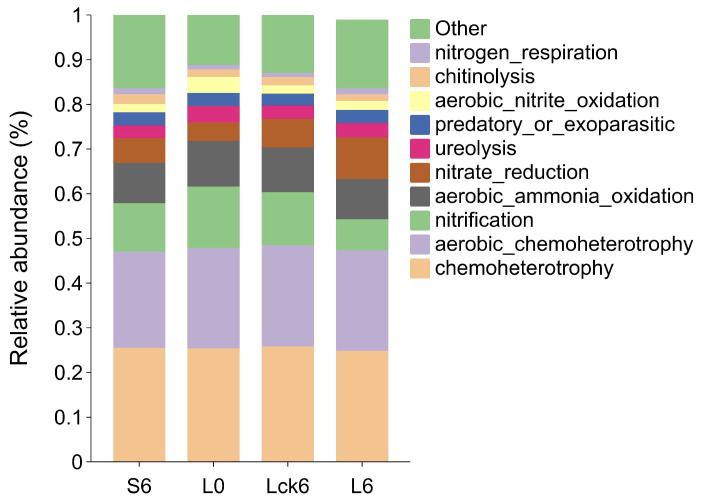
Functional shifts in bacterial metabolism induced by long-term root exudate accumulation. Predicted bacterial metabolic potentials were annotated using FAPROTAX, a database coupling prokaryotic taxa to established ecological functions based on cultivated strains and experimentally validated literature. Comparative analysis revealed that long-term root exudate amendment significantly suppressed nitrification potential while enhancing nitrate reduction capacity compared to the pre-treatment baseline community. Sample abbreviations follow definitions in Figure 1 and Figure 2.

According to FAPROTAX functional prediction, long-term root exudate amendment resulted in a significant decrease in nitrification potential compared to the sterile water control. Furthermore, when compared to the pre-exudate baseline community, the exudate-treated microbiome showed not only reduced nitrification but also enhanced nitrate reduction capacity (Figure 4).

These findings indicate that sustained root exudation reprograms the nitrogen cycle in the rhizosphere, shifting its balance toward reductive pathways. The observed suppression of nitrification coupled with stimulation of nitrate reduction suggests a metabolic rerouting that may ultimately promote the stepwise reduction of nitrate to gaseous forms. Such a shift could drive direct nitrogen loss from the soil system, simultaneously disrupting microbial nutrient cycling and compromising plant nitrogen availability.

This functional reprogramming highlights a mechanistic link between root exudate accumulation and nitrogen depletion in monoculture soils that a cascade that likely contributes to the progressive decline in soil fertility and plant performance observed in consecutive monoculture systems.

### 3.5. Root Exudate Accumulation Reshapes Bacterial Taxonomic Composition

To dissect the specific bacterial taxa responsive to root exudate accumulation, we performed detailed taxonomic analysis across treatment groups. Our findings reveal that exudate application drives a significant reorganization of the rhizosphere bacterial assemblage, characterized by the selective enrichment of a subset of taxa and the suppression of several potentially beneficial microorganisms.

**Figure 5:**
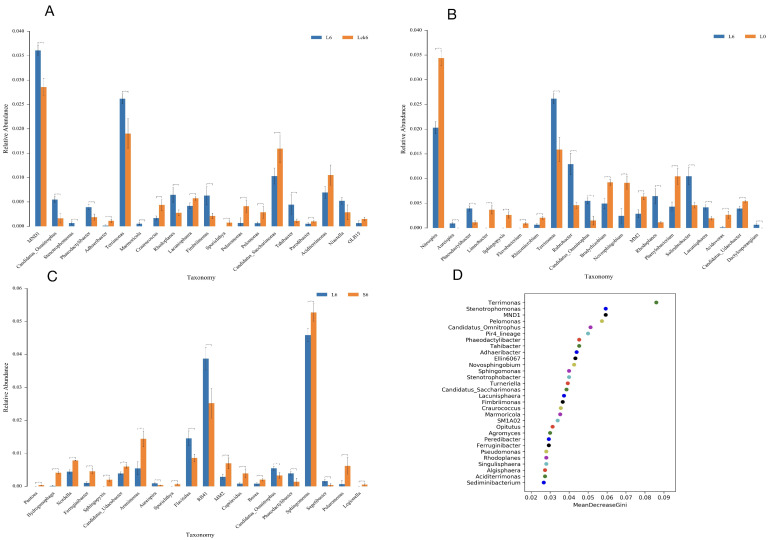
Root exudate accumulation reshapes bacterial taxonomic composition. (**A**) Differentially abundant bacterial genera in the rhizosphere following long-term root exudate amendment compared to sterile water control. (**B**) Bacterial taxa demonstrating significant abundance shifts between long-term exudate amendment and the pre-treatment baseline community. (**C**) Comparative analysis of rhizosphere bacterial composition under long-term versus short-term root exudate exposure. (**D**) Random forest regression identifying the most important bacterial genera characterizing the long-term exudate-amended rhizosphere. Sample abbreviations follow definitions in Figure 1 and Figure 2. * *p* < 0.05, ** *p* < 0.01 (two-tailed Student’s *t*-test).

Exudate amendment enriches specific bacterial genera. Compared to the sterile water control, root exudate application significantly increased the relative abundance of MND1 (to 3.5%, *p* < 0.01) and *Terrimonas* (to 2.5%, *p* < 0.01) (Figure 5A).

Exudates suppress nitrifying and plant-associated bacteria while promoting oligotrophic taxa. Relative to the pre-treatment baseline, exudate exposure induced a pronounced decline in the nitrifying genus *Nitrospira* (from 3.4% to 2%, *p* < 0.01). Furthermore, multiple bacterial genera with documented roles in nutrient cycling and plant interactions, including *Limnobacter*, *Sphingopyxis*, *Flavobacterium*, *Rhizomicrobium*, *Bradyrhizobium*, *Novosphingobium*, MM2, *Phenylobacterium*, *Acidovorax*, and *Candidatus udaeobacter*, were significantly depleted to near-undetectable levels (<0.01%). Conversely, we observed marked enrichment of *Terrimonas* (2.7%), *Rubrobacter*, *Candidatus omnitrophus*, *Rhodoplanes*, and *Solirubrobacter* (all exceeding 0.05%) (Figure 5B).

Long-term accumulation favors a distinct bacterial profile. When comparing long-term versus short-term exudate treatments, the majority of differentially abundant genera exhibited declining trends, with the notable exception of genus RB41, which increased significantly to 3.8% (Figure 5C).

Machine learning identifies key exudate-responsive taxa. Random forest analysis ranked the following genera as the most important biomarkers for long-term root exudate exposure: *Terrimonas*, *Stenotrophomonas*, MND1, *Pelomonas*, *Candidatus omnitrophus*, Pir4 lineage, *Phaeodactylibacter*, *Tahibacter*, *Adhaeribacter*, and Ellin6067 (Figure 5D).

Our taxonomic profiling demonstrates that root exudate accumulation acts as a strong selective force, driving the rhizosphere bacterial community toward a specialized state dominated by taxa adapted to exudate-rich conditions. The consistent enrichment of *Terrimonas* and the decline of nitrifying and plant-growth-promoting genera suggest a functional restructuring that may compromise nitrogen transformation and plant–microbe mutualisms. The identified key taxa represent potential biological indicators for monitoring soil health in monoculture systems and provide candidate targets for future microbiome engineering aimed at mitigating replant disease.

### 3.6. Bacterial Taxa Associated with the Soil-Borne Pathogen Fusarium

Understanding the bacterial taxa that interact with the soil-borne pathogen *Fusarium* is essential for deciphering interspecies dynamics in the rhizosphere, constructing synthetic biocontrol communities, and appreciating the complexity of native soil ecosystems.

**Figure 6:**
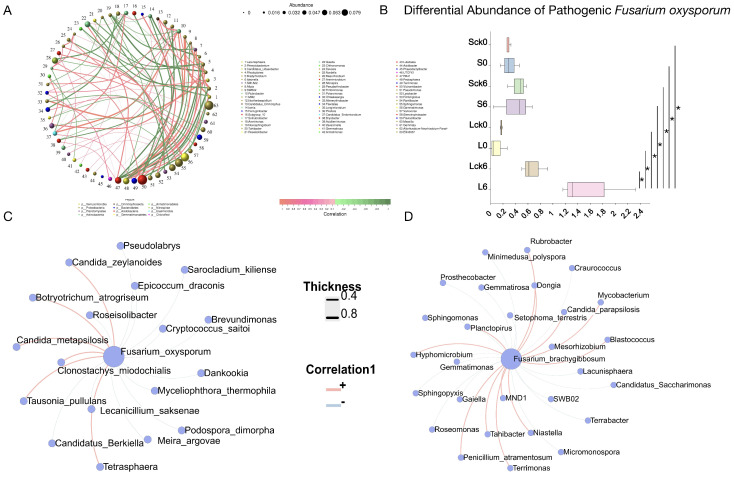
Bacterial taxa associated with the soil-borne pathogen *Fusarium*. (**A**) Bacterial taxa showing significant correlations in the rhizosphere under long-term root exudate amendment compared to sterile water control. (**B**) Relative abundance of *F. oxysporum* across treatments, demonstrating its specific enrichment in long-term exudate-amended soils. * *p* < 0.05. (**C**) Taxa exhibiting significant negative correlations with *F. oxysporum* across all treatment samples, identified using SparCC sparse correlation analysis ( |r| > 0.8, *p* < 0.05). (**D**) Bacterial genera demonstrating significant negative correlations with *F. brachygibbosum*, providing potential candidates for constructing synthetic biocontrol communities.

Analysis of the bacterial community under long-term root exudate accumulation identified several highly connected and abundant keystone taxa, including *Vicinamibacter*, Ellin6067, *Sphingomonas*, and *Gemmatimonas*, suggesting their potential role in maintaining microbial network structure and function (Figure 6A).

Relative abundance of *Fusarium oxysporum* across treatments (Figure 6B), demonstrating its specific enrichment in long-term exudate-amended soils compared to sterile water controls, untreated, and short-term exudate groups. This indicates that sustained root exudation is a critical factor driving pathogen enrichment and ultimately contributes to negative plant–soil–microbe feedback and replant disease.

Additionally, we found that the pathogenic *Fusarium oxysporum* was negatively associated with *Brevundimonas*, *Roseisolibacter*, *Cryptococcus saitoi*, *Dankookia*, *Lecanicillium saksenae*, *Myceliophthora thermophila*, and *Candidatus Berkiella* (Figure 6C).

We further identified specific bacterial genera exhibiting significant correlations with *F. brachygibbosum*. The genera *Terrimonas*, MND1, and *Tahibacter* were all enriched under long-term exudate treatment, and they showed a significant positive correlation with the pathogen. In contrast, multiple bacterial taxa were negatively correlated with *F. brachygibbosum*, including *Craurococcus*, *Gaiella*, *Gemmatimonas*, *Candidatus saccharimonas*, *Gematimorosa*, *Mesorhizobium*, and *Sphingomonas* (Figure 6D).

These correlation patterns delineate a network of potential facilitative and antagonistic interactions between the rhizosphere bacterial community and key fungal pathogens. The negatively correlated taxa represent a prioritized reservoir of candidates for constructing synthetic communities designed to suppress *Fusarium* proliferation and mitigate root rot in monoculture systems.

## 4. Discussion

*Fusarium* species represent the most prevalent and destructive plant pathogens worldwide [9], driving root rot and consecutive monoculture problems across major crops such as wheat [10] and medicinal plants like *Panax notoginseng* [11], where they can cause complete plantation collapse. Despite their agricultural significance, the mechanisms underlying *Fusarium* enrichment and disease exacerbation remain poorly understood, impeding effective control strategies [12]. Prevailing studies emphasize the beneficial role of root exudates in recruiting protective microbiomes, yet largely overlook their potential role in fostering negative plant–soil feedback [13]. Our work provides compelling evidence that long-term accumulation of root exudates in *P. notoginseng* systems actively enriches *Fusarium* in the rhizosphere, effectively turning the plant’s own chemical signals into a liability. This pathogen-tropic shift underscores that exudate-mediated recruitment must be central to any effective disease management strategy.

Our results indicate that the long-term addition of *Panax notoginseng* root exudates significantly reduces the alpha diversity of the rhizobacterial community. A decline in alpha diversity typically reflects decreased species richness and evenness, which may simplify community structure, reduce niche overlap, and weaken microbial interaction networks [14]. Such simplification can diminish the functional redundancy and stability of the rhizosphere microbiome, thereby compromising its buffering capacity against environmental disturbances or pathogen invasion [15]. Beta diversity analysis further revealed clear separation among treatment groups, suggesting that root exudates not only alter taxonomic diversity but also reshape the overall community architecture. This structural restructuring may involve shifts in the relative abundance of key functional groups or the enrichment/depletion of specific microbial taxa, ultimately affecting the functional equilibrium of the rhizosphere ecosystem. Of particular concern is that reduced bacterial alpha diversity may increase the risk of pathogen colonization. Diverse microbial communities often form a “biological barrier” through nutrient competition, niche exclusion, or the production of antimicrobial compounds, thereby suppressing pathogen establishment and expansion [16]. When community diversity declines, this innate disease-suppressive capacity may be compromised, allowing pathogens to more readily colonize the rhizosphere and initiate infection [17].

Moving forward, the construction of synthetic microbial communities represents a promising and sustainable biocontrol strategy. Our study provides a mechanistic blueprint to guide this endeavor. First, our network analysis identified specific bacterial and fungal taxa that show significant positive or negative correlations with Fusarium abundance. This delineates a candidate pool for informed community design [18]. Second, we have demonstrated that root exudates profoundly reshape the soil microbiome. Consequently, synthetic consortia must incorporate taxa that can either utilize or tolerate these compounds to enable cross-feeding and cooperative pathogen suppression [19]. Finally, it is critical to reconsider plant physiology under monoculture stress—specifically, why plants exude seemingly ‘self-harming’ metabolites. Incorporating beneficial microbes that alleviate such stress may reduce the release of autotoxic compounds. This approach could disrupt the detrimental feedback loop and promote root health in intensive agriculture [20].

This study transcends the specific case of *P. notoginseng* to illuminate a fundamental challenge in modern intensive agriculture [21]. We have identified a self-perpetuating cycle in which domestication practices, specifically continuous monoculture, drive plants to unconsciously engineer their own demise through root exudate-mediated microbiome manipulation. This “ecological betrayal” mechanism represents a paradigm shift in our understanding of replant disease. It moves beyond simple nutrient depletion or pathogen accumulation to reveal a sophisticated ecological dysfunction [22]. Our findings compel a global re-evaluation of sustainable agricultural practices, particularly for high-value perennial crops experiencing similar yield declines. They establish a new conceptual framework in which root exudates are recognized not merely as nutritional substrates but as critical ecological signals amenable to strategic management [23]. By demonstrating how exudate composition determines microbiome assembly and pathogen susceptibility, we provide the scientific foundation for developing next-generation solutions. These include exudate-guided breeding programs, precision microbiome engineering, and targeted amendment strategies, all capable of breaking this destructive cycle [24]. This research ultimately positions plant root exudate management as a cornerstone of ecological agriculture, offering a transformative approach to enhance soil health and global food security while reducing dependence on chemical interventions.

## 5. Conclusions

Our integrated findings demonstrate that long-term root exudate accumulation in *P. notoginseng* monoculture systems drives a specific rhizosphere microbiome reprogramming, characterized by fungal diversity loss, bacterial community restructuring, and selective pathogen enrichment. Furthermore, *Fusarium* spp. respond to specific root exudates, with their community dynamics tied to rhizosphere chemistry. Third, we identify keystone taxa most susceptible to this perturbation, including the enriched *Terrimonas* and MND1, and the suppressed *Nitrospira* and plant-growth-promoting rhizobacteria. Beyond mechanistic insights, our co-occurrence network analysis provides a microbial interaction blueprint for designing targeted biocontrol strategies. By mapping taxa positively and negatively correlated with *Fusarium* abundance, we enable the rational design of synthetic communities that can disrupt pathogen establishment while restoring soil health. These findings transform our understanding of replant disease from a pathogen-centric view to an ecological perspective centered on microbiome management, offering new solutions for sustainable agriculture through manipulation of plant–microbe interactions.

While providing a correlative landscape of microbiome shifts, the amplicon-based approach of this study points to the need for deeper functional validation. Future efforts would benefit from integrating metagenome-assembled genomes (MAGs) to resolve the metabolic potential of key responsive taxa such as Terrimonas and Nitrospira. Further, metatranscriptomics (RNA-seq) could directly link taxonomic restructuring to real-time gene-expression dynamics in the rhizosphere, offering a mechanistic transition from correlation to causation.

## Data Availability

The original contributions presented in the study are included in the article. Further inquiries can be directed to the corresponding authors.
